# An Overview of Health-Promoting Programs and Healthy Lifestyles for Adolescents and Young People: A Scoping Review

**DOI:** 10.3390/healthcare12202094

**Published:** 2024-10-21

**Authors:** Marta Raimundo, Ana Cerqueira, Tania Gaspar, Margarida Gaspar de Matos

**Affiliations:** 1 Aventura Social—Associação, 1649-026 Lisbon, Portugal; martaraimundo2009@hotmail.com (M.R.); tania.gaspar.barra@gmail.com (T.G.); margarida.gaspardematos@gmail.com (M.G.d.M.); 2 ISAMB, Medicine Faculty, Lisbon University, 1649-026 Lisbon, Portugal; 3 HEI-Lab: Digital Human-Environment Interaction Labs, Lusófona University, 1749-024 Lisbon, Portugal; 4 Católica Research Centre for Psychological, Family and Social Wellbeing, Faculty of Human Sciences, Portuguese Catholic University, 1649-023 Lisbon, Portugal; 5 Applied Psychology Research Center Capabilities & Inclusion-APPsyCI, ISPA University Institute, 1149-041 Lisbon, Portugal

**Keywords:** youth programs, healthy lifestyles, mental health, adolescents, young adults

## Abstract

The health of children, adolescents, and young adults is a primary global concern. In 2021, there were 2.1 million deaths among children and adolescents. Injuries, violence, communicable diseases, nutritional deficiencies, substance use, non-communicable diseases, and mental health disorders are among the leading causes of death in this age group. **Background/objectives:** This scoping review aims to identify and describe health promotion and healthy lifestyle programs developed worldwide targeting adolescents and young adults. **Methods:** A total of 106 programs were included, of which 8 were selected through scientific databases and 98 through other research methods (e.g., government websites and other online sources). **Results:** The results show that Europe, North America, New Zealand, and the United States of America are the continents and countries with the highest number of programs. Most programs originated before 2020 and are aimed at children, adolescents, and young adults. Mental health, substance and non-substance dependencies, and sexual and reproductive health were the most frequent areas among the available youth programs. Most programs do not mention evaluating or monitoring the services provided. **Conclusions:** This work allows for a deeper understanding of the programs available for adolescents and young adults, providing an overview of their characteristics. Moreover, it emphasizes the importance of increasing the number of available programs, especially in countries with higher morbidity and mortality rates among the young population. The programs must be based on population studies to better meet their needs. Lastly, programs should become sustainable and integrated into national public policies, accompanied by ongoing training, supervision, and intervision of professionals working in these contexts.

## 1. Introduction

In 2021, there were approximately 2.1 million deaths of children, adolescents, and young people aged 5 to 24 [1]. UNICEF [1] estimates that between 2022 and 2030 the number of deaths for this age group will increase to 19 million. According to the World Health Organization (WHO) [2], the primary causes of death among children and adolescents include injuries (traffic accidents and drowning), suicide, violence, communicable diseases (maternal and perinatal), nutritional deficiencies (e.g., iron deficiency anemia), substance use, non-communicable diseases such as sexually transmitted diseases (e.g., human immunodeficiency virus—HIV), obesity, physical inactivity, and mental health disorders. The most prevalent mental health disorders are depression, anxiety, and behavioral problems in childhood. Although these indicators are global, there is a higher incidence of deaths in certain regions, including East and Southeast Asia, West Asia, Africa, and Oceania (excluding Australia and New Zealand) [1].

The well-known definition of health by the WHO [3] fully justifies its scope and the importance of addressing its determinants. These determinants include individual factors (e.g., age and gender), socioeconomic factors (e.g., access to housing, employment, health services, culture, and education), environmental factors (e.g., air quality, water, and sound), and organizational and political factors (e.g., health, education, and transport systems). These factors can act independently or in groups [4,5]. In addition to these factors, it is essential to mention the impact that the COVID-19 pandemic has had on young people’s development, well-being, and health. On the one hand, many young people have experienced an increase in symptoms of depression, anxiety, and loneliness due to the measures imposed by the pandemic, such as the closure of services and restrictions on social interactions, among others [6,7]. On the other hand, young people also report that the pandemic has contributed to increased leisure time, moments of self-reflection, and connection with others [6].

Health promotion and health-promoting schools, universities, and workplaces are critical concepts that have been repeatedly framed by the WHO [8,9]. In this regard, the WHO outlined a set of guidelines for health interventions and services aimed at the young population. These guidelines reinforce the importance of acting in various areas to promote young people’s physical and mental health and prevent risky behaviors. Therefore, mental health, substance use, sexually transmitted diseases and pregnancy, violence and injuries, nutrition, and physical activity are areas of particular focus in interventions with young people [10].

Access to responses tailored to different populations is essential to promote physical and mental health. One example of this is the implementation of youth programs designed to respond to the needs of young people. These programs can be implemented at international, national, or regional/local levels and aim to provide young people with information, strategies, activities, and health services, considering their needs and characteristics [2]. Access to these services influences their health and well-being and how they will relate and behave in different contexts (e.g., school, home, or a group of friends) [11]. This scoping review aims to identify and describe health promotion and healthy lifestyle programs developed worldwide which target adolescents and young adults. The focus of this scoping review is to understand how these health promotion and lifestyle programs are distributed globally and what their main areas of focus are. Additionally, the study aims to determine whether these programs are specifically designed for the youth population, their year of origin, and whether they are subject to evaluation. In this context, the following research questions are addressed: (1) What are the key features of health promotion and healthy lifestyle programs for adolescents and young adults globally? (2) How are health promotion and healthy lifestyle programs for adolescents and young adults distributed globally?

## 2. Method

**Figure 1:**
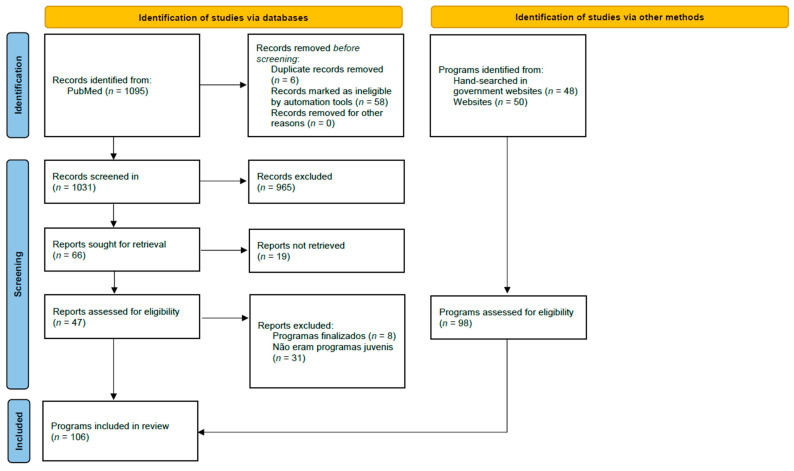
PRISMA flow diagram. Adapt from Page et al. [13].

A scoping review was carried out using the PRISMA checklist guidelines for scoping reviews [12]. The information of the review registered on OSF can be found at Supplementary Materials. The PRISMA flow diagram allows one to observe the obtained programs’ identification, selection, eligibility assessment, and inclusion processes (Figure 1). The research was conducted from March to December 2023. Both scientific databases and gray literature were utilized in this research, which was conducted in multiple languages, including Portuguese, English, French, and Spanish.

First, the PubMed database was searched using the following terms for titles and abstracts: program AND Adolescent OR Youth OR Teenager OR Young people AND Health OR Mental Health AND Promote AND community. A total of 1095 articles were identified, of which 64 were removed (6 duplicate articles and 58 on topics irrelevant to the research objective). After reading the titles and abstracts, 965 articles that did not represent youth programs for health promotion and healthy lifestyles were removed. Of the 66 articles selected for reading, 19 did not have the full text available. After reading the full text of the remaining articles, 39 were excluded: 8 because they addressed completed programs and 31 because they were not programs with accessible responses for young people.

Only PubMed was selected as a scientific search engine, considering the characteristics of the programs, as these services are provided by government and non-profit entities and are not subject to scientific evaluation. Therefore, the research focused on the use of other search engines (e.g., Google) and government websites. Forty-eight programs were identified on government websites and fifty on other websites. Thus, 106 youth programs for promoting health and healthy lifestyles were included.

The programs were included in this study if they (1) had active services at the time of the research, (2) contained websites, articles, or reports available for consultation, (3) were aimed directly or indirectly at adolescents and/or young adults, and (4) promoted physical and mental health and healthy lifestyles.

### Data Analysis

The first author examined the 106 selected programs and extracted the following data: program name, year of inception, country, continent, responsible entity, national or regional intervention, area(s) of intervention, target population, objectives, description of activities/services, if it includes evaluation, and website URL/source. The extracted content was entered into a database for further analysis.

The data were analyzed using the Statistical Package for Social Sciences (SPSS) version 29. This tool was employed to conduct frequency analysis and characterize the youth programs.

## 3. Results

A total of 106 youth health promotion and healthy lifestyle programs were identified: eight scientific articles, 98 websites, and reports from other research sources. Table 1 lists the programs selected for this scoping review.

Based on the distribution of youth programs across different continents, 40% of the programs were located in Europe (*n* = 53), 21.7% in North America (*n* = 23), and 17.0% in South America (*n* = 18). Oceania (*n* = 9), Asia (*n* = 2), and Africa (*n* = 1) had the fewest youth programs promoting health and healthy lifestyles (Table 2).

Our research on the distribution of youth programs across countries has yielded significant findings. New Zealand emerged with the highest number of programs, boasting nine, followed closely by the United States of America with eight. Canada, Germany, Belgium, and France each had six programs (Table 3), while Mexico, Brazil, Spain, and Norway had five. Chile, Ecuador, Austria, Italy, the United Kingdom, and Portugal had four programs, and Colombia had three. The countries with the fewest programs were Argentina, Bulgaria, Poland, and Czechia, with two programs each, and South Africa, Costa Rica, Honduras, Panama, the Dominican Republic, Bolivia, Peru, Uruguay, Republic of Korea, Hong Kong, Cyprus, and the Netherlands had one program each. It is important to note that these results only represent the countries included in the study, as the language barrier made it difficult to obtain information on the existence of health promotion and well-being programs in other countries.

Regarding the year of inception, 82.0% of the programs began by 2020 (*n* = 50), while 18.0% started after 2020 (*n* = 11). This year was marked by the COVID-19 pandemic, which led to the closure of various services worldwide. In this context, it is important to highlight the emergence of several services during and after the pandemic, most of which were forced to adapt their services to comply with the restrictions imposed by social confinement [14]. As for the target population, most programs (96.2%) are aimed at adolescents and young adults (see Table 4).

Table 5 shows the distribution of programs concerning each of the areas mentioned. Mental health received the highest number of responses from youth programs (86.8%), followed by addictions (43.4%) and sexual and reproductive health (35.8%). Physical activity and nutrition received the lowest responses from youth programs (24.5% and 22.6%, respectively).

Finally, in terms of program evaluation, the majority do not mention having an evaluation (61.3%), while 38.7% mention that they carry out some evaluation (*n* = 41) (Table 6).

## 4. Discussion

This scoping review aimed to identify and characterize health and healthy lifestyle promotion programs developed worldwide for adolescents and young adults. The research was conducted from March to December 2023. One-hundred-and-six youth health and healthy lifestyle promotion programs were included, of which eight were selected through scientific databases and ninety-eight through other research sources.

Most programs for adolescents and young people are developed in Europe, North America, and South America. In contrast, Oceania, Asia, and Africa have fewer programs. This fact is concerning because Africa bears a high incidence of deaths among children and young people [1], which underscores the pressing health issues faced by these age groups.

Despite several public policy measures being implemented in recent years, the stark reality of mortality rates reinforces the urgent need to increase responses and intervention services for this age group. Effective interventions targeted at adolescents and young people promote health and well-being, reduce future complications, and reduce infant and youth mortality [10].

With the COVID-19 pandemic, there was an increase in anxiety, depression symptoms, and loneliness among the young population [6]. However, due to the closure of services and the restrictions imposed during this period, there was a need to improve the available responses to meet emerging needs. This new reality led several countries to feel the need to create, adapt, or expand their services to respond to the population’s needs [15,16]

As the return to normality unfolded, a diverse range of responses in service delivery was observed. Some services discontinued their remote assistance, while others continued or adopted a combination of both [16]. This diversity was also evident in this study. Despite only 18.0% of the identified programs being created after 2020 (*n* = 11), many other programs adjusted their services, such as providing support remotely via telephone or chat.

The programs most frequently mention mental health, substance abuse and addictions, and sexual and reproductive health, in line with the WHO’s guidelines that reinforce the importance of working in these areas with this population [10]. However, although the WHO also mentions other action areas, such as nutrition and physical activity, these services were less frequently mentioned in the identified programs.

The literature emphasizes that interventions for young people should aim to promote physical and mental health, encourage physical activity, ensure adequate and healthy nutrition, and prevent addictions and risky sexual behaviors [2,17,18,19]. Over the past decade, sleep hygiene has gained prominence, with its impact on the health of adolescents and young people increasing alongside the rise in screen use [20,21]. This study reveals that the included programs address at least one key area, with mental health, addictions (both with and without substance use), and sexual and reproductive health being the most frequently covered topics. It is worth mentioning that a Good Practices platform is also available in Portuguese on a website associated with Direção Geral de Estatísticas da Educação e Ciência—DGEEC (Directorate General of Education and Science Statistics) [22].

In conclusion, providing responses tailored to the needs of young people is essential to promote their physical and mental health and a healthy lifestyle. These programs should be implemented considering the population’s characteristics and needs [2,10]. In some countries, health promotion takes place in a school setting as part of the educational program. However, the responses are often insufficient given the high number of students and the lack of human resources [23]. In this regard, it is crucial to increase the number of available programs outside the school setting, as well as their accessibility, especially in countries that both offer few programs and have a high number of deaths in this age group due to physical health issues (e.g., injuries, violence, nutritional deficiencies, and non-communicable diseases) and mental health issues (e.g., depression, anxiety, and behavioral problems).

## 5. Conclusions

This work enabled a global analysis of health promotion and healthy lifestyle programs for adolescents and young adults. The distribution analysis across continents and countries provides an overview of the available responses, revealing a prevalence of programs in developed countries and a limited number in other regions. Examining covered areas, target populations, and the presence of program evaluation offers insight into the characteristics of the youth programs included in this review.

The results show that Europe, North America, and South America are the continents with the highest number of programs. New Zealand, the United States of America, Canada, Germany, Belgium, and France were the countries with the most programs. Regarding the origin of the programs, the vast majority started before 2020 and are aimed directly at children, adolescents, and young people. Mental health, substance and non-substance addictions, and sexual and reproductive health were the most frequent areas among available youth programs. Most programs do not mention any evaluation or monitoring of services provided.

Some limitations of this study must be considered. Information from sources such as government and other websites is often only available in the native language. This restricts access to information, as the documents may not be available in any of the languages used in this scoping review. Additionally, not all articles were available for consultation, which, due to the financial and time constraints of the project, prevented other procedures from being carried out to obtain the full texts. Another limitation of the study is that only active programs were included, as the aim was to provide an overview of the services currently available to young people. As a result, scientific articles based on programs implemented for research purposes were not included, since these are usually carried out over a short period and do not offer ongoing services to the target population. Lastly, it is important to note that most programs provide little information on the type of assessment or monitoring of the services offered, which limits a more in-depth analysis of this indicator. Program assessment is crucial for making necessary adjustments to meet the needs and characteristics of the target population. Therefore, youth programs must implement assessment and monitoring methods to ensure the quality of their services.

This study underscores the importance of increasing the number of available programs, particularly in countries with the highest incidence of morbidity and mortality among the young population. It further emphasizes the need for these programs to be based on population studies, as this is crucial for accurately identifying and addressing the evolving needs of young people. For instance, the growing needs related to managing screen time, social media, online gaming, sleep hygiene, and mental health, especially during and after the COVID-19 pandemic, can be effectively addressed through evidence-based approaches.

This study also emphasizes the need for programs to become sustainable and integral to national public policies. Programs should move away from being temporary, episodic, and non-evaluated and instead adopt a scientific approach in their implementation and evaluation. Finally, enhancing the training, supervision, and intervision of the professionals who implement and promote these programs is crucial.

## Figures and Tables

**Table 1:** Youth health promotion and healthy lifestyle programs.

Project Name	Year of Inception	Country	Sexual and Reproductive Health	Mental Health	Addictive Behaviors and Dependencies	Eating Behaviour	Physical Activity	Specific for Young People?	Evaluation	Target Population	Website URL
1737	--	New Zealand	No	Yes	No	No	No	No	--	Teenagers/young adult	https://1737.org.nz/ (accessed on 15 June 2023)
988 Suicide & Crisis Lifeline	2005	USA	No	Yes	No	No	No	No	--	Teenagers/young adult	https://988lifeline.org/ (accessed on 5 May 2023)
Academia de Lideres Ubuntu (Ubuntu Leadership Academy)	2010	Portugal	No	Yes	No	No	No	Yes	Yes	Teenagers/young adult	https://academialideresubuntu.org/pt/escolas-ubuntu (accessed on 6 March 2023)
Active Children Through Incentive Vouchers—Evaluation (ACTIVE)	--	The Netherlands	No	No	No	No	Yes	Yes	Yes	Teenagers/young adult	https://doi.org/10.1136/bmjopen-2018-025618 (accessed on 6 July 2023)
Activilandia	2013	Spain	No	No	No	Yes	Yes	Yes	--	Teenagers/young adult	https://activilandia.aesan.gob.es/ACTIVILANDIA/index.html (accessed on 14 April 2023)
Adolescent Health Programme Student Health Service	2001	Hong Kong	Yes	Yes	Yes	Yes	Yes	Yes	Yes	Teenagers/young adult	https://www.ahpshs.gov.hk/english/abo_us/abo_us.html (accessed on 14 September 2023)
Agenda Gap	--	Canada	Yes	Yes	No	No	No	Yes	Yes	Teenagers/young adult	https://kdehub.ca/projects/mhp-if/promoting-adolescent-mental-health-through-policy-refining-and-testing-a-multi-level-intervention-to-promote-individual-and-population-mental-health-through-youth-engaged-policymaking-2/ (accessed on 31 October 2023)
Allianz für Gesundheitskompetenz (Alliance for Health Literacy)	2017	Germany	No	Yes	Yes	No	Yes	No	--	Teenagers/young adult	https://www.bundesgesundheitsministerium.de/gesundheitskompetenz/allianz-fuer-gesundheitskompetenz.html (accessed on 10 November 2023)
Amigas Latinas Motivando el Alma	2011	USA	No	Yes	No	No	No	No	Yes	--	https://doi.org/10.1007/s11414-017-9582-7 (accessed on 12 December 2023)
Anamatas	--	New Zealand	Yes	Yes	No	No	No	Yes	Yes	Teenagers/young adult	https://www.anamata.org.nz/ (accessed on 15 June 2023)
Aquí estoy (I’m here)	--	Costa Rica	No	Yes	No	No	No	No	--	Teenagers/young adult	https://aquiestoy.cr/ (accessed on 29 September 2023)
Beratungs und Therapiezentrum (Counseling and Therapy Center)	2018	Belgium	No	Yes	No	No	No	No	--	Teenagers/young adult	https://www.btzentrum.be/ (accessed on 13 October 2023)
Bittelebe (Please leave)	--	Austria	No	Yes	No	No	No	Yes	--	Teenagers/young adult	https://bittelebe.at/ (accessed on 14 September 2023)
Bundeszentrale für gesundheitliche Aufklärung (Federal Center for Health Education)	2008	Germany	Yes	Yes	Yes	Yes	Yes	No	Yes	Teenagers/young adult	https://www.bzga.de/ (accessed on 5 May 2023)
CeGIDD—Centres gratuits d’information, de dépistage et de diagnostic (Free information, screening, and diagnostic centers)	--	France	Yes	No	No	No	No	No	--	Teenagers/young adult	https://www.auvergne-rhone-alpes.ars.sante.fr/centres-gratuits-dinformation-de-depistage-et-de-diagnostic-vih-ist-hepatite-c-0 (accessed on 14 April 2023)
Centros Juveniles del Ayuntamiento de Madrid (Youth Centers of the Madrid City Council)	--	Spain	No	Yes	No	No	No	Yes	--	Teenagers/young adult	https://www.madrid.es/portales/munimadrid/es/Inicio/Jovenes/Direcciones-y-telefonos/Centros-Juveniles-del-Ayuntamiento-de-Madrid/?vgnextfmt=default&vgnextoid=ae68e4bf4ae67310VgnVCM2000000c205a0aRCRD&vgnextchannel=fe07b7dd3f7fe410VgnVCM1000000b205a0aRCRD (accessed on 10 November 2023)
Contacto Joven (Young Contact)	--	Mexico	No	Yes	No	No	No	Yes	--	Teenagers/young adult	https://mexico.ureport.in/page/contactojoven/ (accessed on 12 December 2023)
CPZ—Centrum ter Preventie van Zelfdoding (Center for Suicide Prevention)	1979	Belgium	No	Yes	No	No	No	No	Yes	Teenagers/young adult	https://www.preventiezelfdoding.be/ (accessed on 13 October 2023)
CVV—Centro de Valorização da Vida (Life Appreciation Center)	1962	Brazil	No	Yes	No	No	No	No	Yes	Teenagers/young adult	https://www.cvv.org.br/ (accessed on 14 April 2023)
dusevnizdravi.edu.cz	--	Czechia	No	Yes	No	No	No	Yes	--	Teenagers/young adult	https://dusevnizdravi.edu.cz/ (accessed on 17 November 2023)
EDU Project	--	Italy	No	No	Yes	No	No	No	--	--	http://www.drogaedu.it/ (accessed on 14 April 2023)
Envolve	2004	New Zealand	Yes	Yes	No	No	No	Yes	Yes	Teenagers/young adults	https://www.evolveyouth.org.nz/ (accessed on 14 April 2023)
Espaces Santé Jeunes (Youth Health Spaces)	1997	France	Yes	Yes	Yes	Yes	No	Yes	--	Teenagers/young adults	https://www.fesj.org/ (accessed on 5 May 2023)
EVRAS—Education à la Vie Relationelle, Affective et Sexuelle (Education in Relationships, Affective and Sexual Life)	2012	Belgium	Yes	No	No	No	No	Yes	--	Teenagers/young adults	https://www.evras.be/ (accessed on 13 October 2023)
Feel-ok.at	--	Switzerland, Austria, and Germany	Yes	Yes	Yes	Yes	Yes	Yes	--	Teenagers/young adults	https://www.feel-ok.at/de_AT/jugendliche/jugendliche.cfm (accessed on 10 November 2023)
Fonoinfancia	--	Chile	No	Yes	No	No	No	Yes	Yes	Teenagers/young adults	https://www.fonoinfancia.cl/quienes-somos/ (accessed on 12 December 2023)
Fortaleça sua Saúde (Strengthen your Health)	2014	Brazil	No	No	Yes	No	Yes	Yes	Yes	Teenagers/young adults	https://doi.org/10.4025/jphyseduc.v31i1.3125 (accessed on 24 March 2023)
Foundry	--	Canada	Yes	Yes	Yes	Yes	Yes	Yes	Yes	Teenagers/young adults	https://foundrybc.ca/ (accessed on 5 May 2023)
Fundación ANAR (ANAR Foundation)	1970	Spain and Latin America	Yes	Yes	Yes	No	No	Yes	--	Teenagers/young adults	https://www.anar.org/ (accessed on 17 November 2023)
Guadagnare Salute: rendere facili le scelte salutari (Gaining Health: Making Healthy Choices Easy)	2007	Italy	No	No	Yes	Yes	Yes	No	--	Teenagers/young adults	https://www.basilicatainsalute.it/guadagnare-salute (accessed on 24 March 2023)
Gut Drauf—Bewegen, Essen, Entspannen (In a good mood—move, eat, relax)	2003	Germany	No	Yes	No	Yes	Yes	Yes	Yes	Teenagers/young adults	https://www.gutdrauf.net/ (accessed on 14 April 2023)
Hablemos de todo (Let’s talk about everything)	--	Chile	Yes	Yes	Yes	Yes	Yes	Yes	--	Teenagers/young adults	https://hablemosdetodo.injuv.gob.cl/ (accessed on 17 November 2023)
Healthpoint	--	New Zealand	No	Yes	No	No	No	No	--	Teenagers/young adults	https://www.healthpoint.co.nz/ (accessed on 24 March 2023)
Helseutvalget (The Health Committee)	1983	Norway	Yes	Yes	Yes	No	No	No	Yes	Teenagers/young adults	https://www.helseutvalget.no/ (accessed on 14 April 2023)
Ich bin alles (I am everything)	2021	Germany	No	Yes	No	No	No	Yes	Yes	Teenagers/young adults	https://www.ich-bin-alles.de/ (accessed on 15 June 2023)
IN FORM	2008	Germany	No	No	No	Yes	Yes	No	Yes	Teenagers/young adults	https://www.in-form.de/ (accessed on 10 November 2023)
Jongeren advies centrum—JAC (Youth Advice Center)	1960	Belgium	Yes	Yes	Yes	No	No	Yes	--	Teenagers/young adults	https://www.caw.be/jac/ (accessed on 13 October 2023)
Kaiser Permanente	1950	USA	Yes	Yes	Yes	Yes	Yes	No	Yes	--	https://community.kp.org/ (accessed on 12 December 2023)
Kaleido—Zentrum für die gesunde Entwicklung von Kindern und Jugendlichen (Kaleido—Center for the Healthy Development of Children and Adolescents)	2018	Belgium	Yes	Yes	No	No	No	Yes	--	Teenagers/young adults	https://www.kaleido-ostbelgien.be/ (accessed on 13 October 2023)
Katie Brown Educational Program	2001	USA	No	Yes	No	No	No	Yes	Yes	Teenagers/young adults	https://kbep.org/ (accessed on 12 December 2023)
Kids Help Phone	1989	Canada	No	Yes	Yes	No	No	Yes	Yes	Teenagers/young adults	https://kidshelpphone.ca/ (accessed on 31 October 2023)
Kirkens SOS (The Church’s SOS)	1974	Norway	No	Yes	No	No	No	No	Yes	Teenagers/young adults	https://www.kirkens-sos.no/ (accessed on 14 April 2023)
Kors på halsen (Cross on the neck)	--	Norway	Yes	Yes	No	No	No	Yes	--	Teenagers/young adults	https://rodekors.service-now.com/x/tnrc/korspahalsen/index (accessed on 24 March 2023)
KYS—Käpiti Youth Support	1998	New Zealand	Yes	Yes	Yes	No	No	Yes	--	Teenagers/young adults	https://kys.org.nz/ (accessed on 20 June 2023)
Leczenie e-uzależnień u dzieci (Treating e-addiction in children)	2021	Poland	No	Yes	Yes	No	No	Yes	--	Teenagers/young adults	https://pacjent.gov.pl/aktualnosc/leczenie-e-uzaleznien-u-dzieci-pilotaz (accessed on 6 July 2023)
Línea 141 (Line 141)	--	Colombia	No	Yes	Yes	No	No	Yes	--	Teenagers/young adults	https://www.icbf.gov.co/noticias/linea-141 (accessed on 29 September 2023)
Línea de apoyo emocional (Emotional support line)	2020	Uruguay	No	Yes	No	No	No	No	--	Teenagers/young adults	https://www.asse.com.uy/contenido/Linea-de-apoyo-emocional--0800-1920-12295 (accessed on 6 July 2023)
Línea de ayuda para la niñez (Helpline for children)	--	Honduras	No	Yes	No	No	No	Yes	--	Teenagers/young adults	https://dinaf.gob.hn/2021/11/25/dinaf-pone-a-disposicion-de-las-ninas-y-ninos-linea-de-denuncias-gratuita-110/ (accessed on 20 June 2023)
Línea de la vida (Life line)	--	Mexico	No	Yes	Yes	No	No	Yes	--	Teenagers/young adults	https://www.gob.mx/salud/conadic/acciones-y-programas/centro-de-atencion-ciudadana-contra-las-adicciones-134381 (accessed on 31 October 2023)
Línea Familia Segura (Safe Family Line)	2020	Bolivia	Yes	Yes	Yes	No	No	No	Yes	Teenagers/young adults	https://www.unicef.org/bolivia/familia-segura (accessed on 22 December 2023)
Línea Familiar (Family Line)	--	Dominican Republic	No	Yes	No	No	No	No	--	Teenagers/young adults	https://lineafamiliar.do/ (accessed on 31 October 2023)
Línea Libre-1515 (Free Line-1515)	2019	Chile	No	Yes	No	No	No	Yes	Yes	Teenagers/young adults	https://www.linealibre.cl/ (accessed on 17 November 2023)
Línea SalvaVidas 24/7 (Lifeguard Line 24/7)	2015	Colombia	No	Yes	No	No	No	Yes	--	Teenagers/young adults	https://www.sergiourrego.org/que-hacemos/#save-lifes (accessed on 12 December 2023)
LINEA106 (Line 106)	1997	Colombia	No	Yes	No	No	No	No	--	Teenagers/young adults	http://www.saludcapital.gov.co/Paginas2/Linea106-Inicio.aspx (accessed on 22 December 2023)
Linha 117 (Line 117)	--	Ecuador	No	Yes	No	No	No	No	--	Teenagers/young adults	https://www.salud.gob.ec/a-traves-de-la-linea-171-msp-ofrece-atencion-en-salud-mental/ (accessed on 6 July 2023)
Llama a la vida (Call for life)	--	Spain	No	Yes	No	No	No	Yes	--	Teenagers/young adults	https://www.sanidad.gob.es/en/linea024/home.htm (accessed on 6 March 2023)
Mapa de contenidos relacionados con Jóvenes (Map of contents related to Youth)	--	Spain	No	No	Yes	Yes	Yes	Yes	--	Teenagers/young adults	https://estilosdevidasaludable.sanidad.gob.es/mapaWebGrupos.do?grupo=Jovenes (accessed on 6 March 2023)
Mapa Saúde Mental (Mental Health Map)	2013	Brazil	Yes	Yes	Yes	Yes	Yes	No	--	Teenagers/young adults	https://mapasaudemental.com.br/ (accessed on 24 March 2023)
Mental Helse Ungdom (Mental Health Youth)	--	Norway	Yes	Yes	Yes	Yes	Yes	Yes	--	Teenagers/young adults	https://mentalhelseungdom.no/ (accessed on 12 December 2023)
MINDBASE-Tools	2019	Austria	No	Yes	Yes	No	No	No	Yes	Teenagers/young adults	https://mindbase.at/ (accessed on 20 June 2023)
Molde Kommune (Molde Municipality)	--	Norway	Yes	Yes	Yes	Yes	No	Yes	--	Teenagers/young adults	https://www.molde.kommune.no/helse-og-omsorg/psykisk-helse/tilbud-til-ungdom/ (accessed on 22 December 2023)
National Youth Program	--	Bulgaria	Yes	Yes	Yes	Yes	Yes	Yes	--	Teenagers/young adults	https://nism.bg/en/ (accessed on 14 September 2023)
Nevypusť duši (Don’t let go of your soul)	2015	Czechia	No	Yes	No	No	No	Yes	--	Teenagers/young adults	https://nevypustdusi.cz/ (accessed on 22 December 2023)
NUBE, Nucleo Urbano de Bienestar Emocional (NUBE, Urban Nucleus of Emotional Well-being)	--	Mexico	No	Yes	Yes	No	No	Yes	--	Teenagers/young adults	https://www.ryapsicologos.net/wp-content/uploads/2021/08/nube.pdf (accessed on 6 July 2023)
PAPYRUS	1997	United Kingdom	No	Yes	No	No	No	Yes	--	Teenagers/young adults	https://www.papyrus-uk.org/ (accessed on 17 November 2023)
Plan Enia	--	Argentina	Yes	No	No	No	No	Yes	--	Teenagers/young adults	https://www.argentina.gob.ar/salud/plan-enia (accessed on 22 December 2023)
Pode falar (He can talk)	2021	Brazil	Yes	Yes	Yes	No	No	Yes	--	Teenagers/young adults	https://www.podefalar.org.br/ (accessed on 24 March 2023)
Points d’accueil et d’écoute jeunes—PAEJ (Youth reception and listening points)	--	France	Yes	Yes	Yes	No	No	Yes	--	Teenagers/young adults	https://solidarites.gouv.fr/points-accueil-et-ecoute-jeunes-paej-0 (accessed on 20 June 2023)
Por ti: Programa de Promoção de Bem-estar Mental nas Escolas (For you: Mental Well-being Promotion Program in Schools)	2022	Portugal	No	Yes	No	No	No	Yes	Yes	Teenagers/young adults	https://www.zurich.com.pt/pt-pt/a-zurich/sala-de-imprensa/comunicados-imprensa/2022/27-set (accessed on 6 March 2023)
PREPARE	2010	Africa	Yes	No	No	No	No	Yes	Yes	Teenagers/young adults	https://bmcpublichealth.biomedcentral.com/articles/10.1186/1471-2458-14-54 (accessed on 12 May 2023)
Progetto CCM (CCM Project)	2018	Italy	No	Yes	Yes	No	No	Yes	--	Teenagers/young adults	https://www.epicentro.iss.it/scuola/progetto-uso-consapevole-internet-giochi (accessed on 29 September 2023)
Program Młodzieżowych Liderów Profilaktyki Uzależnień i Promocji Zdrowia (Addiction Prevention and Health Promotion Youth Leaders Program)	--	Poland	Yes	Yes	Yes	Yes	Yes	Yes	Yes	Teenagers/young adults	https://www.tppu.org/programy/4-program-mlodziezowych-liderow-profilaktyki-uzaleznien-i-promocji-zdrowia (accessed on 24 March 2023)
Programa Cuida-te+ (Take Care + Program)	2011	Portugal	Yes	Yes	Yes	Yes	Yes	Yes	Yes	Teenagers/young adults	https://ipdj.gov.pt/o-programa (accessed on 6 March 2023)
Programa de Atención Psicológica a Distancia (Remote Psychological Care Program)	--	Mexico	No	Yes	No	No	No	No	--	Teenagers/young adults	https://saludmental.unam.mx/psicologia-3.htm (accessed on 5 May 2023)
Programma di promozione della salute mentale nelle scuole (Mental Health Promotion Program in Schools)	2010	Italy	No	Yes	No	No	No	Yes	--	Teenagers/young adults	https://www.epicentro.iss.it/mentale/defObiettivi (accessed on 24 March 2023)
Programme national de lutte contre le tabac (National Tobacco Control Program)	2023	France	No	No	Yes	No	No	No	--	Teenagers/young adults	https://sante.gouv.fr/actualites/actualites-du-ministere/article/un-nouveau-programme-national-de-lutte-contre-le-tabagisme-2023-2027 (accessed on 20 June 2023)
Projeto Dream Teens (Dream Teens Project)	2014	Portugal	Yes	Yes	Yes	Yes	Yes	Yes	Yes	Teenagers/young adults	https://gulbenkian.pt/en/projects/inquerito-literacia-saude-portugal-2/ (accessed on 6 March 2023)
PsicólogosxChile (PsychologistsxChile)	--	Chile	No	Yes	No	No	No	No	--	Teenagers/young adults	https://psicologosxchile.cl/ (accessed on 12 May 2023)
Rat auf Draht (Advice on the wire)	1987	Austria	No	Yes	No	No	No	Yes	Yes	Teenagers/young adults	https://www.rataufdraht.at/ (accessed on 20 October 2023)
Red de escucha (Listening network)	--	Peru	No	Yes	No	No	No	Yes	--	Teenagers/young adults	https://www.lacasadelafamilia.pe/red-de-escucha (accessed on 14 September 2023)
Salud Mental es cosa de Todas y Todos (Mental Health is Everyone’s Business)	2023	Argentina	Yes	Yes	Yes	Yes	No	No	--	Teenagers/young adults	https://www.argentina.gob.ar/jefatura/instituto-nacional-de-juventud/salud-mental (accessed on 12 May 2023)
SAPTEL	1994	Mexico	No	Yes	No	No	No	No	--	Teenagers/young adults	https://www.saptel.org.mx/index.html (accessed on 29 September 2023)
Services de Santé Étudiante (Student Health Services)	--	France	Yes	Yes	Yes	Yes	Yes	Yes	--	Teenagers/young adults	https://santetudiant.com/ (accessed on 10 March 2023)
Shout	--	United Kingdom	No	Yes	No	No	No	Yes	Yes	Teenagers/young adults	https://www.crisistextline.uk/ (accessed on 22 December 2023)
StopBlues	2018	France	No	Yes	No	No	No	No	Yes	Teenagers/young adults	https://www.inserm.fr/actualite/stopblues-site-et-appli-pour-prevenir-mal-etre/ (accessed on 10 March 2023)
Sucht & Drogen Hotline (Drugs & Addiction Hotline)	2003	Germany	No	No	Yes	No	No	No	--	Teenagers/young adults	https://www.sucht-und-drogen-hotline.de/wirueberuns/index.html (accessed on 10 November 2023)
SUPRA	--	Austria	No	Yes	No	No	No	No	--	Teenagers/young adults	https://www.gesundheit.gv.at/leben/suizidpraevention.html (accessed on 20 October 2023)
Taiohi Türama	1993	New Zealand	Yes	Yes	No	No	No	Yes	--	Teenagers/young adults	https://taiohiturama.org.nz/ (accessed on 22 December 2023)
Teen Talk	--	Canada	Yes	Yes	Yes	No	No	Yes	--	Teenagers/young adults	https://teentalk.ca/ (accessed on 5 May 2023)
The Jed Foundation	1998	USA	No	Yes	Yes	No	No	Yes	Yes	Teenagers/young adults	https://jedfoundation.org/ (accessed on 12 December 2023)
The UK Youth Fund—Thriving Minds	--	United Kingdom	No	Yes	No	No	No	Yes	Yes	--	https://www.ukyouth.org/thriving-minds/ (accessed on 17 November 2023)
The Youth Mental Health Project	--	USA	No	Yes	No	No	No	Yes	--	Teenagers/young adults	https://ymhproject.org/ (accessed on 20 June 2023)
Tía Elaine	2020	Panama	No	Yes	No	No	No	Yes	Yes	Teenagers/young adults	https://senniaf.gob.pa/?page_id=16121 (accessed on 22 December 2023)
Topity	2021	Brazil	No	Yes	No	No	No	Yes	Yes	Teenagers/young adults	https://www.unicef.org/brazil/topity-um-chatbot-para-melhorar-sua-autoestima (accessed on 10 March 2023)
Vibe	1996	New Zealand	Yes	Yes	No	No	No	Yes	--	Teenagers/young adults	https://www.vibe.org.nz/ (accessed on 12 May 2023)
Vlaams Instituut Gezond Leven (Flemish Institute for Healthy Living)	1991	Belgium	No	Yes	Yes	Yes	Yes	No	Yes	Teenagers/young adults	https://www.gezondleven.be/ (accessed on 20 October 2023)
Wellness Together Canada	2020	Canada	No	Yes	Yes	No	No	No	--	Teenagers/young adults	https://www.wellnesstogether.ca/en-ca/ (accessed on 31 October 2023)
Whangarei Youth Space	--	New Zealand	Yes	Yes	No	No	Yes	Yes	--	Teenagers/young adults	http://whangareiyouthspace.co.nz/ (accessed on 12 December 2023)
Young Leaders Council	--	USA	No	Yes	No	No	No	Yes	Yes	Teenagers/young adults	https://mhanational.org/young-leaders/council (accessed on 12 December 2023)
YoungMinds	--	United Kingdom	No	Yes	No	No	No	Yes	Yes	Teenagers/young adults	https://www.youngminds.org.uk/ (accessed on 12 December 2023)
Youth Mental Health Canada	2013	Canada	No	Yes	No	No	No	Yes	Yes	Teenagers/young adults	https://ymhc.ngo/ (accessed on 31 October 2023)
Youth One Stop Shops (YOSS)	1994	New Zealand	Yes	Yes	Yes	No	Yes	Yes	--	Teenagers/young adults	https://www.yoss.org.nz/ (accessed on 12 December 2023)
Youth.gov	--	USA	Yes	Yes	Yes	Yes	Yes	Yes	Yes	Teenagers/young adults	https://youth.gov/ (accessed on 20 June 2023)
Περσέας (Perseus)	--	Cyprus	No	No	Yes	No	No	Yes	--	Teenagers/young adults	https://shso.org.cy/clinic/kentro-symvouleftikis-efivon-kai-oikogeneias-perseas/ (accessed on 17 November 2023)
Haциoнaлнa пpoгpaмa зa пpeвeнция нa хpoничнитe нeзapaзни бoлecти (National Program for the Prevention of Chronic Non-Communicable Diseases)	2017	Bulgaria	No	Yes	Yes	Yes	Yes	No	Yes	Teenagers/young adults	https://ncpha.government.bg/index/3189-nacionalna-programa-za-prevencia-na-hronichnite-nezarazni-bolesti-2021-2025-g.html?lang=en (accessed on 20 October 2023)
한국청소년상담복지개발원 (Korea Youth Counseling & Welfare Institute)	1993	Republic of Korea	No	Yes	Yes	No	No	Yes	--	Teenagers/young adults	https://www.kyci.or.kr/userSite/index.asp (accessed on 12 May 2023)

**Table 2:** Distribution of youth programs by continent.

	*n*	%
Europe	53	40.0
North America	23	21.7
South America	18	17.0
Oceania	9	8.5
Asia	2	1.9
Africa	1	0.9

**Table 3:** Distribution of adolescent and youth programs by country.

	*n*	%
**Africa**		
South Africa	1	0.9
**North America**		
Canada	6	5.7
Costa Rica	1	0.9
United States of America	8	7.5
Honduras	1	0.9
Mexico	5	4.7
Panama	1	0.9
Dominican Republic	1	0.9
**South America**		
Argentina	2	1.9
Bolivia	1	0.9
Brazil	5	4.7
Chile	4	3.8
Colombia	3	2.8
Ecuador	4	3.8
Peru	1	0.9
Uruguay	1	0.9
**Asia**		
Republic of Korea	1	0.9
Hong Kong	1	0.9
**Europe**		
Germany	6	5.7
Austria	4	3.8
Belgium	6	5.7
Bulgaria	2	1.9
Cyprus	1	0.9
Spain	5	4.7
France	6	5.7
Italy	4	3.8
Norway	5	4.7
Nertherlands	1	0.9
Poland	2	1.9
Portugal	4	3.8
United Kingdom	4	3.8
Czechia	2	1.9
Switzerland, Austria, and Germany	1	0.9
**Oceania**		
New Zealand	9	8.5

**Table 4:** Distribution of programs for adolescents and young people by year of inception and target population.

	*n*	%
**Year of inception**		
Untill 2020	50	82.0
After 2020	11	18.0
**Target population**		
Teenagers/young adult	102	96.2
Parents, teachers, and other professionals who work with young people	4	3.8

**Table 5:** Distribution of youth programs by area of intervention.

	Yes		No	
	*n*	%	*n*	%
Sexual and reproductive health	38	35.8	68	64.2
Mental health	92	86.8	14	13.2
Addictions	46	43.4	60	56.6
Nutrition	24	22.6	82	77.4
Physical activity	26	24.5	80	75.5

**Table 6:** Distribution of programs for adolescents and young people by type of evaluation.

	*n*	%
Yes	41	38.7
Does not mention	65	61.3

## Data Availability

Data sharing is not applicable to this article as no datasets were generated or analysed during the current study.

## Supplementary Materials

The following supporting information can be downloaded at: https://www.mdpi.com/article/10.3390/healthcare12202094/s1, This scoping review adheres to the Preferred Reporting Items for Systematic Reviews and Meta-Analyses extension for Scoping Reviews (PRISMA-ScR) checklist [12]. The information of the review registered on OSF can be found at: https://doi.org/10.17605/OSF.IO/YHZKW.
